# Potential of Molecular Weight and Structure of Tannins to Reduce Methane Emissions from Ruminants: A Review

**DOI:** 10.3390/ani9110856

**Published:** 2019-10-23

**Authors:** Isaac A. Aboagye, Karen A. Beauchemin

**Affiliations:** Lethbridge Research and Development Centre, Agriculture and Agri-Food Canada, 5403 1st Avenue South, Lethbridge, AB T1J 4B1, Canada; iaboagye@ualberta.ca

**Keywords:** tannins, methane, ruminants, performance

## Abstract

**Simple Summary:**

Regardless of the production system adopted, ruminant livestock contribute to greenhouse emissions that are associated with climate change. Among the greenhouse gases, enteric methane produced from the rumen is of the greatest concern because it is the largest single source of livestock emissions. Among the different dietary strategies examined to decrease methanogenesis in ruminants, the use of tannins shows promise, but has received only moderate attention. However, tannins are abundant in both tropical and temperate plants and so are widely available globally and may be an economical approach for livestock producers to mitigate enteric methane emissions. This review explores the challenges and opportunities of using dietary tannins to reduce enteric methane emissions from ruminants.

**Abstract:**

There is a need to reduce enteric methane (CH_4_) to ensure the environmental sustainability of ruminant production systems. Tannins are naturally found in both tropical and temperate plants, and have been shown to consistently decrease urinary nitrogen (N) excretion when consumed by ruminants. However, the limited number of in vivo studies conducted indicates that the effects of tannins on intake, digestibility, rumen fermentation, CH_4_ production and animal performance vary depending on source, type, dose, and molecular weight (MW). There are two main types of tannin in terrestrial plants: condensed tannin (CT; high MW) and hydrolysable tannin (HT; low MW). Consumption of CT and HT by ruminants can reduce N excretion without negatively affecting animal performance. High MW tannins bind to dietary protein, while low MW tannins affect rumen microbes, and thus, irrespective of type of tannin, N excretion is affected. The structure of high MW tannin is more diverse compared with that of low MW tannin, which may partly explain the inconsistent effects of CT on CH_4_ production reported in in vivo studies. In contrast, the limited number of in vivo studies with low MW HT potentially shows a consistent decrease in CH_4_ production, possibly attributed to the gallic acid subunit. Further in vivo studies are needed to determine the effects of tannins, characterized by MW and structural composition, on reducing CH_4_ emissions and improving animal performance in ruminants.

## 1. Introduction

Ruminants occupy the largest area of agricultural land worldwide and are efficient in using fibrous feeds that cannot be used as human food. Ruminants contribute to food security, especially in developing countries with growing populations. However, the environmental sustainability of ruminant production systems has been highly criticized because ruminants contribute to greenhouse gas (GHG) emissions that are implicated in climate change [1]. Methane (CH_4_) is the largest source (44%) of GHG emissions in the lifecycle of ruminant production and is mainly from enteric fermentation [2]. Methane emissions also account for 6% to 12% of energy intake in ruminants [3], representing a potential inefficiency.

Tannins have been examined largely for their role in endo-parasite control and improving nitrogen (N) utilization of ruminants. About 10% to 40% of consumed N is retained as meat or milk by ruminants [4], with the majority of dietary N excreted in feces and urine. Excretion of N contributes to ammonia (NH_3_) and nitrous oxide emissions that have negative impacts on the environment. Forage diets are often high in soluble crude protein (CP) content, which exacerbates the situation by increasing the proportion of N (40% to 75%) excreted in the highly labile form of urine [5]. Feeding tannins to ruminants improves N utilization by decreasing rumen degradability of CP and sometimes CP digestibility in the total digestive tract, which shifts N excretion from urine to feces and consequently, reduces excretion of the more volatile form of N into the environment [6]. This effect may be independent of source, type, molecular weight (MW) or dose of tannin [7,8].

Tannins may also play a role in mitigating methanogenesis. In vitro studies have shown that tannins have anti-methanogenic activity, either directly by inhibiting methanogens or indirectly by targeting protozoa [9,10]. The effects of tannins on in vivo CH_4_ reduction appear to depend on the source, subunit, MW and dose. Jayanegara et al. [11] showed in a meta-analysis study that the reduction in CH_4_ production expressed on the basis of digestible organic matter (OM) intake was highly variable when tannin concentration was < 2.0 g/100 g of dietary dry matter (DM). All the in vivo experiments in that meta-analysis used condensed tannin (CT)-containing forages or extracts, with the exception of one study that used hydrolysable tannin (HT) extract [12]. It is evident that past in vivo research on the effect of tannins has focused mainly on CT with inconsistent effects on CH_4_ reduction. Moreover, high MW tannin is structurally more diverse and complex relative to low MW tannin [13] and therefore, differing effects of HT and CT when used in ruminant diets to decrease CH_4_ production are inevitable. For instance, isolated CT from a natural plant source is estimated to contain about 22 billion distinct chemical entities when the subunits and linkages of CT are taken into consideration [14]. This complexity may account for the inconsistent effects of CT on enteric CH_4_ production [15]. Since HT have low MW and are less structurally variable than CT, they appear to result in a more consistent CH_4_ reduction effect. The effect of HT on reducing enteric CH_4_ production may be due to the gallic acid (GA) subunit [8], although few in vivo studies have characterized the effect of low MW tannins on CH_4_ production. Herein, we review the current literature on the potential of CT and HT for decreasing CH_4_ production while considering their effects on animal performance. 

## 2. Production and Mitigation of Enteric Methane

Ruminants rely on a consortia of microbes under anaerobic conditions of the rumen to degrade plant structural carbohydrates (cellulose and hemicellulose), proteins and other organic polymers into monomers. The monomers are then fermented to end-products such as volatile fatty acids (VFA), NH_3_, carbon dioxide (CO_2_), and dihydrogen (H_2_). The VFA (primarily acetate, propionate and butyrate) are used by the animal as a main source of energy, while CO_2_ and H_2_ and sometimes formate are used by some methanogens (e.g., *Methanomicrobiales, Methanopyrales, Methanococcales, Methanobacteriales, Methanocellales and Methanosarcinales;* [16,17]) to form CH_4_. Other substrates, such as methyl compounds (methanol, mono, di and tri-methylamine), can also be used by some archaea (e.g., *Methanoplasmatales* or *Thermoplasmatales*-related archaea; [16,17,18]) to form CH_4_. The formation of CH_4_ as a sink for H_2_ highlights the importance of methanogens to rumen microbial fermentation and indirectly to plant fibre digestion [19]. During glycolysis, intercellular cofactors such as Reduced Nicotinamide Adenine Dinucleotide (NADH) need to be re-oxidized (NAD^+^) for fermentation to continue enabling microbial growth [20]. The NADH is oxidized through H_2_ production, but this process is thermodynamically less competitive at elevated partial pressure of H_2_ in the rumen. Methanogens utilize H_2_ to reduce CO_2_ to CH_4_, thereby keeping the partial pressure of H_2_ low to enable cofactors to be re-oxidized for continuous microbial fermentation [18]. This process optimizes the digestion of plant fibre; however, eructation of CH_4_ from the rumen represents a loss of energy (13.3 Mcal/kg CH_4_). 

Numerous strategies have been explored as a means of decreasing enteric CH_4_ emissions from ruminant animals, with many comprehensive reviews published (e.g., [21]). Despite the extensive amount of research, few CH_4_ mitigation approaches are available for immediate adoption by producers, other than sustainable intensification of livestock production [22]. Adoption of mitigation strategies are at different levels of acceptance due to uncertainties in effectiveness, lack of information on animal production and additional costs of implementation. Theoretically, a decline in enteric CH_4_ production should result in a greater amount of metabolizable energy available to the animals and consequently, greater net energy for production if the efficiency of converting metabolizing energy to net energy for weight gain or milk production is not altered and if dry matter intake (DMI) and digestibility are not negatively affected. However, when H_2_ is not used to reduce CO_2_ to CH_4_ some of the alternative H_2_ sinks in the rumen cannot be used as energy substrates by animals (e.g., formate or gaseous H_2_). Thus, it is possible for enteric CH_4_ to be decreased without improvement in weight gain or milk production. 

Thus, CH_4_ mitigation options that are inexpensive and simultaneously ensure efficient use of energy are needed. Such mitigation efforts would not only lessen the economic burden to farmers and consumers but would allow wide implementation to reduce enteric CH_4_ emissions associated with ruminant production. The use of tannins may offer such a possibility because they are naturally occurring in numerous plants, and hence widely available to ruminant producers. 

## 3. Sources of Tannin and Global Perspectives 

### 3.1. Sources and Chemical Diversity of Tannins

**Figure 1:**
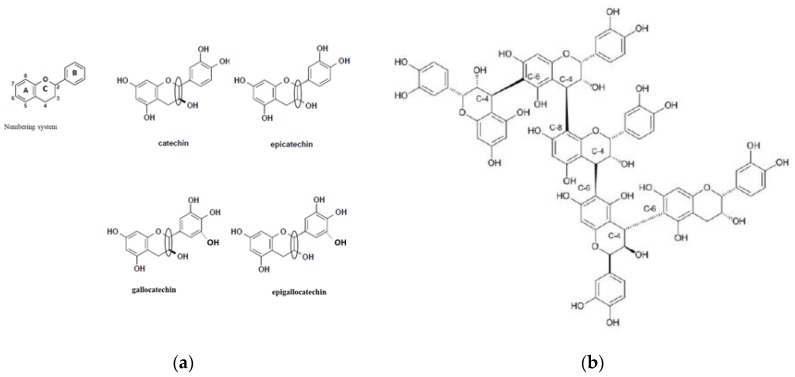
Subunits and interlinkage structures of flavan-3-ols occurring in condensed tannins, described as: (**a**) Procyanidin (catechin and epicatechin) and prodelphinidin (gallocatechin and epigallocatechin) condensed tannin subunits; and (**b**) 4,8- and 4,6-B-type interflavan linkage in condensed tannin oligomers and polymers. Source [14,26].

Tannins are a class of polyphenol (hydroxyl attached to aromatic rings) compounds. The large number of phenolic hydroxyl groups enables tannins to react mainly with protein and to a lesser extent with carbohydrates [23]. Based on the reactivity and structural characteristics of tannins, they are generally grouped as CT, HT and phlorotannins (PT). The CT and HT are found in terrestrial plants, while PT is only found in marine algae (e.g., red and brown algae [24]). Terrestrial tannins are extensively distributed in the plant kingdom and are abundant in many forages, shrubs, cereals and medicinal herbs. The CT are also known as proanthocyanidins, consisting of oligomers or polymers of flavan-3-ol subunits [14,25]. They have high MW of 1900 to 28,000 Da and their subunits differ due to the hydroxyl groups and the relative stereochemistry (spatial orientation) of the C-2 and C-3 ring (Figure 1; circled). The most common ones are procyanidin (e.g., catechin and epicatechin, which upon oxidation gives rise to cyanidin) and prodelphinidin subunits (e.g., gallocatechin and epigallocatechin, both are products of delphinidin upon oxidation). The bonding patterns of CT subunits into oligomers and polymers occur mainly through covalent linkages of the C-4 position of the C-ring of one flavan-3-ol to mainly the C-8 and C-6 positions in the C-ring of other subunits (Figure 1; B-type linkages, 4–8 and 4–6; [14]).

**Figure 2:**
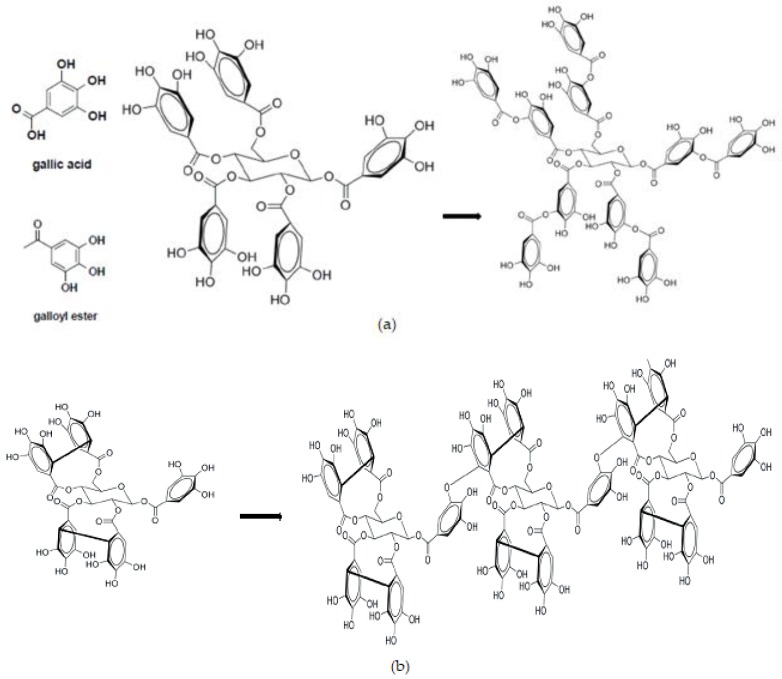
Subunits and interlinkage structures of gallotannin and ellagitannin in hydrolysable tannins, described as (**a**) β-1,2,3,4,5,6-pentagalloyl glucose forming gallotannin (tannic acid) and (**b**) casuarictin (ellagitannin) forming trimer of casuarictin (ellagitannin). Source [14,26].

Hydrolysable tannin has relatively low MW (500 to 3000 Da) and unlike CT, is usually made up of a glucose core, although it may contain other core molecules (glucitol, hammamelose, shikimic acid, quinic acid, and quercitol), with hydroxyl groups esterified with GA. Thus, HT are derivatives of GA. Further esterification and oxidative cross-linkages on the galloyl group result in the formation of additional HT (Figure 2; [14,26]). The HT can be divided into two major subclasses: gallotannins and ellagitannins. Gallotannins are formed when GA units are added to the galloyl groups. This type of HT is commonly referred to as tannic acid (TA). Through intramolecular oxidative coupling, the galloyl group is dimerized forming ellagic acid moieties. The coupling can be between adjacent GA such as the galloyl groups on glucose C-4 and C-6 (eugeniin) or C-2 and C-3 (casuarictin; also has C-4 and C-6). The casuarictin in turn, may form other intermolecular bonds with itself (e.g., trimer casuarictin) or with gallotannins.

**Figure 3:**
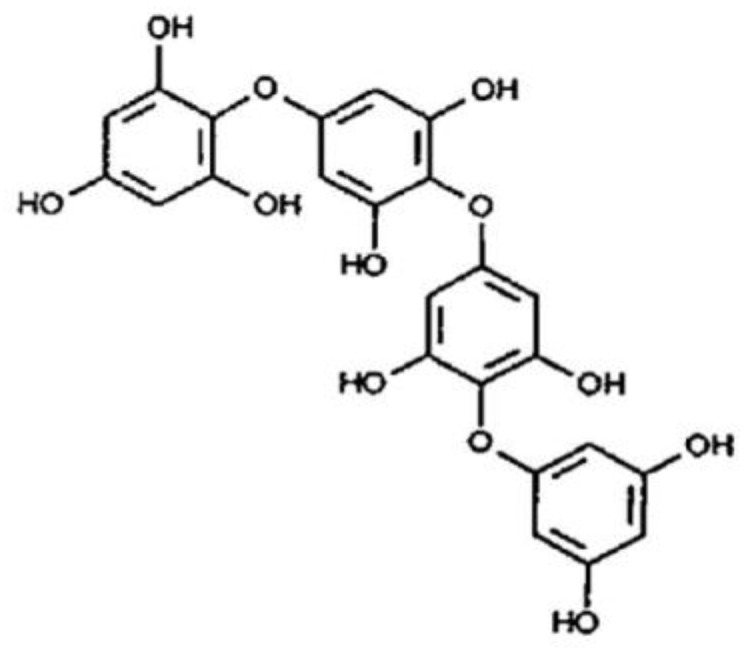
Model structure of phlorotannins. Source [24].

The PT are formed as a result of the polymerization of phloroglucinol (1,3,5-trihydroxybenzene) and have a MW of 126 to 650,000 Da (Figure 3; [24]). However, PT are structurally less complex than terrestrial tannins (HT and CT) and can be classified into six categories (phlorethols, isofuhalos, echole, fucole, fuhalols, and fucophlorethols). They are mainly synthesized via the acetate-malonate pathway [27], although other pathways such as the shikimate or the phenylpropanoid pathways have been proposed.

### 3.2. Global Perspectives for Tannin-Containing Feeds

Tannin-containing terrestrial plants are common in many ruminant-grazing areas. In temperate regions, tannins are usually found in forage legumes such as birdsfoot trefoil (*Lotus corniculatus*), greater birdsfoot trefoil (*Lotus pedunculatus*), common vetch (*Vicia sativa*), purple prairie clover (*Dalea purpurea*), sainfoin (*Onobrychis coronarium*), and sulla (*Hedysarum coronarium*). In tropical regions, tannins are commonly found in many leguminous and non-leguminous leaves of trees or shrubs (e.g., *Acacia angustissima*, *Argania spinosa* and *Ceratonia silique*) that are fed to ruminants. 

Condensed tannin is the most common type of tannin in some temperate (range; 0.04 to 9.9 g/100 g DM) and tropical forage legumes (0.7 to 23.8 g/100 g DM), whereas HT (7.6 to 13.9 g/100 g DM) is mainly found in various tropical forages (Table 1). Both types of tannin may be present at different concentrations, depending on the part of the plant, stage of growth, and growing conditions [28]. Generally, tannin concentration is greater in tropical plants relative to temperate plants. This effect is partly due to the effects of drought and warm conditions of tropical regions on chemical composition of the plant. For instance, Top et al. [29] showed that the green leaf of *Quercus rubica* exposed to warm conditions produced 50% more tannins when grown in dry conditions compared with wet conditions (12.0 vs. 8.0 g/100 g). This higher concentration suggests a defensive role of tannin in plants that are environmentally stressed. In addition, the same authors [29] reported that under warm, dry conditions, the tannins produced in *Quercus rubica* were less polymerized compared with wet conditions. This result may partly explain the higher concentration of low MW tannin (i.e., HT) in some tropical plants. However, an increase in tannin concentration dilutes the primary nutritional composition of the plant and decreases the energy content. Thus, a high concentration of tannin can negatively affect the plane of nutrition of ruminants, especially in tropical regions where tannin concentrations are relatively high. 

The challenge for temperate tannin-containing legumes is their low yield relative to non-tannin containing legumes. For instance, sainfoin (1.6 to 9.4 g/ 100 g CT) has a low persistence in cold environments (e.g., western Canada) relative to alfalfa (a non-tannin containing forage). The recent development of hardier cultivars has helped expand the use of sainfoin, although sainfoin experiences winter kill in certain locations making alfalfa a forage of choice for producers [30]. Thus, in many temperate areas, there are few tannin-containing forages, especially those containing HT that can be grown competitively and preserved in a cost-effective manner. 

Extracts are an alternative means of providing tannins to ruminants that are fed formulated diets such as dairy cows and feedlot cattle. Tannin extracts from plants (e.g., mimosa, quebracho and chestnut) are mainly produced on a commercial scale for use by the leather and wine industries [31]. Tannin extracts are, to some extent, in a pure form with uniform chemical composition, unlike tannins in plants that are not uniformly distributed and vary with plant growth. 

## 4. Effects of Tannins in Ruminant Nutrition 

The lack of global standardization of techniques for analysing tannin purity, content and structure, makes it difficult to pinpoint the effects of tannin classification and functionality on animal variables. Thus, future research is needed to improve analytical methodology and characterization of tannins in relation to physiological responses of animals.

### 4.1. Binding Effects of Tannins

The phenolic hydroxyl groups present in tannins allow them to bind with numerous macromolecules, particularly proteins and, to a lesser extent, with carbohydrates, nucleic acids and metal ions [46]. These interactions with other molecules determine the metabolic effects of tannins in the animal. Tannin-protein interactions are the most important determinant of the nutritional value and potential toxicity of tannins in ruminants (Table 2). In the gastrointestinal tract of the ruminant, the tannin-protein complex is usually reversible if it is a non-covalent bond (hydrogen and hydrophobic; [47]). The protein in the complex may be from dietary, microbial, mucous or endogenous sources. The variable pH within the gastrointestinal tract can influence the reversible reaction between tannin and protein, thereby influencing the effect of feeding tannin to animals.

#### 4.1.1. Negative Effects of Tannins

In the past, tannins were considered anti-nutritive when present in feeds because of their potentially negative effects on intake, digestion and absorption of nutrients and ultimately, animal performance [55]. Tannin-containing plants can be less palatable due to the binding of tannin to salivary glycoproteins resulting in an unpleasant taste for the animal [56]. The binding properties of tannins may also decrease fibre digestibility by inhibiting fibre-degrading enzymes or by binding to dietary carbohydrates and in turn, decreasing rumen turnover rate, which can negatively impact intake and animal performance [28,46]. Moreover, high concentrations of tannins (i.e., >5.0 g/100 g DM) may be toxic to the animal by causing irritation and desquamation of the intestinal mucosa, liver and kidneys lesions, ulcers and even death [57]. The anti-nutritive properties and toxicity of tannins are mostly attributed to ingestion of high concentrations of HT because of its poorer adsorption to protein and subsequent release of metabolites in the rumen causing cellular damage [58]. However, CT may also affect intestinal organs [46] and decrease intake and digestibility of proteins such that animal performance is negatively affected [6]. Therefore, it is evident that the negative impacts of tannins on ruminants are not specific to tannin type, but may depend upon the concentration of tannin in the forage or extract. 

#### 4.1.2. Beneficial Effects of Tannins

Supplying a low to moderate concentration to tannin (i.e., <3.0 to 5.0 g/100 g DM) through tannin-rich forages and extracts can have beneficial effects for ruminants by preventing bloat, improving N utilization, decreasing CH_4_ production, acting as an antioxidant, controlling endo-parasites, and improving animal and wool growth and milk production [28,59,60]. Feeding tannin-containing forages to animals with high protein requirements may improve performance due to a potentially greater supply of metabolizable protein to the lower tract as a result of a decrease in degradability of protein. Under such situations, 8% to 38% increases in average daily gain and 10% to 21% increases in milk production, relative to non-tannin containing forages, have been reported [28]. Factors related to the intrinsic characteristics of tannin-containing forages, such as type, digestibility and overall diet quality, can confound the effects of tannin on animal performance. Furthermore, it is very difficult to quantify the effects of tannins on animal performance because the tannin characteristics are confounded with the chemical composition and nutritional value of the plant. The use of tannin extracts as feed supplements can help overcome this limitation to some extent. Free tannin may bind with salivary proteins (e.g., proline) and allow tannin to pass through the digestive tract in a bound form, preventing its degradation, absorption, or interaction with other dietary or endogenous protein [61,62]. On the other hand, free tannins may bind to dietary soluble proteins, decrease rumen NH_3_ concentration, increase the flow of rumen undegraded protein to the lower tract and shift N excretion from urine to faeces, thereby decreasing the volatile form of N excreted into the environment. Free tannins may also target methanogenic microbes or protozoa associated with methanogens and disrupt their activities in the rumen to reduce enteric CH_4_ production in ruminants [9]. 

### 4.2. Rumen Fermentation and Enteric Methane Production (in Vitro and in Vivo)

Under anaerobic conditions of the rumen, tannins may be degraded by microbes into metabolites that affect microbial fermentation and subsequently VFA concentration in the rumen [7,63]. Using tannin-extract from chestnut (HT) or quebracho (CT) as the only carbon source in a culture technique, certain microbes, including *Bacillus pumilus*, *B. polymyxia*, *Klebsiella planticola*, *Cellulomonas*, *Arthrobacter*, *Micrococcus*, *Corynebacterium*, and *Pseudomonas* were shown to produce enzymes that degrade tannins [64]. In the rumen, microbes that utilize tannins degrade them into their subunits that are subsequently converted through the dihydrophloroglucinol and the 3-hydroxy-5-oxohexanoate pathways to acetate and butyrate [65,66] to generate energy.

#### 4.2.1. Mode of Action

Tannins act as rumen modifiers, but the main mechanism by which they affect methanogenesis has not definitively been demonstrated in vitro or in vivo. There are multiple hypotheses of how tannins decrease CH_4_ production: (1) tannins act directly on methanogens [67,68]; (2) they affect protozoa that are associated with methanogens [9]; (3) tannins act on fibrolytic bacteria and decrease fibre degradation [69], and (4) they act as a H_2_ sink [70]. Tannins may function via all, some, or any of the proposed mechanisms, because in studies where significant effects of tannins on CH_4_ abatement have been reported, there has been a large range (in vitro = 4.3% to 70% and in vivo = 6.0% to 68%; Table 3 and Table 4, respectively) in CH_4_ decrease. It is likely that the mechanisms by which tannins reduce CH_4_ production differ with tannin type (MW, source or subunit), concentration, dietary substrate and animal type.

#### 4.2.2. The Mechanistic Effect of MW, Source and Subunit Interactions on *in Vitro* CH_4_ Reduction 

It is generally assumed that the greater the MW, the greater the binding ability of tannin. This MW effect was demonstrated in an in vitro study where oligomeric CT fractions from the legume *Lotus pendunculatus* were inactive against methanogens and did not reduce CH_4_ production compared with polymeric fractions [80]. Similarly, Saminathan et al. [76] reported that greater MW fractions of CT were more efficient in reducing the total population of methanogens than lower MW fractions of CT. On the contrary, Jayanegara et al. [10] showed that HT, which has a lower MW and higher binding ability than CT, decreased the methanogen population and microbes, providing H_2_ to a greater extent than CT. For CT, those that are galloylated (i.e., CT containing GA or galloyl groups at the C-3 position) have a higher binding capacity and precipitate protein more than the non-galloylated forms [13]. Nauman et al. [15] showed that the ability of CT to bind and precipitate protein is not directly related to the inhibition of CH_4_ production, and thus, it appears that the potential of tannins to reduce the methanogen population in the rumen cannot be solely attributed to the ability to bind to methanogens. It is possible that tannins penetrate archaeal cells causing toxicity [67]. This effect may be greater for low MW tannin. In support of this theory, Saminathan et al. [76] showed that the most abundant archaeal community (rumen cluster C; *Thermoplasmatales*-related group) decreased with decreasing MW of CT, although total methanogens increased. It appears non-galloylated CT with high MW are not able to penetrate the cells of some methanogens and cause toxicity. However, low MW tannins with GA derivatives or galloylated CT, which, upon degradation, produces GA, may have selective antimethnogenic effects. For example, Rira et al. [75] reported that a HT-rich forage was 26% more effective in suppressing methanogenesis in vitro than CT-rich sources.

#### 4.2.3. Effects of MW, Source and Subunit Interactions on *in Vivo* CH_4_ Reduction

Most animal studies (Table 4) have largely focused on CT rather than HT and few studies have examined the effects of MW of tannin on animal performance and CH_4_ production. Recently, Stewart et al. [81] compared forages containing HT or CT, while Aboagye et al. [8] compared different sources and forms of HT. The previous focus on CT rather than HT stems from the potential toxic effects of the lower MW HT following hydrolysis in the gastrointestinal tract of the animal, but negative effects of HT can be avoided by gradual adaptation and continuous feeding [88] or using lower concentrations (i.e., <5.0 g/100 g DM [8,85]). However, due to the different analytical methods for quantifying HT, the optimum dose of HT is not known.

Hydrolysable tannin may act directly on rumen microbes because of its lower MW, especially methanogens [10]. This effect may be more pronounced for HT metabolites than the complex forms of HT [8]. However, if HT decrease CH_4_ by binding and/or penetrating the cell of methanogens thereby causing toxicity as has been suggested [67], they may also directly interfere with fibrolytic bacteria. There is also the possibility that a decrease in methanogens would increase the partial pressure of H_2_ in the rumen with negative effects on fibre degradation. However, recent in vivo studies with chemo-inhibitors have shown 20% to 40% decreases in CH_4_, 600-fold increases in gaseous H_2_ emissions, but no negative effects on animal production [89]. Furthermore, tannins may also act as a H_2_ sink [70] to prevent the negative feedback of reduced cofactors on fibre degrading microbes. Thus, a reduction in CH_4_ production when feeding HT does not necessarily imply negative effects on animal performance.

A recent study compared feeding CT-containing hay [birdsfoot trefoil (0.6 g/100 g CT) or sainfoin (2.5 g/100 g CT)], HT-containing hay [small burnet (4.5 g/100 g HT)] or non-tannin containing hay (alfalfa, cicer milkvetch, or meadow bromegrass) to heifers (DM basis). The HT-containing hay decreased CH_4_ emission (g/day) by about 25% compared with both the CT-containing and non-tannin-containing hays [85]. This result was largely due to lower DMI for heifers fed the HT-containing hay, as CH_4_ yield (g/kg DMI) was similar for all tannin-containing forages. It is not clear whether the lower DMI was due to the presence of HT, but digestibility was likely not a factor as neutral detergent fibre digestibility was actually greater for the HT-containing hay than for the other tannin-containing forages. In the same study, the HT-containing hay decreased CH_4_ yield of beef cows by 39% relative to those fed the CT-containing hay diets, with no difference in intake. These contrasting results for heifers and cows may indicate that HT has the potential to inhibits methanogenesis but effects may depend on the intake level of the cattle. Aboagye et al. [7] reported that HT combined with CT (50:50; 1.5 g/100 g tannin in the dietary DM) added to a high forage diet decreased CH_4_ emissions without negatively affecting the growth of beef cattle compared with the control cattle (no tannin). Nevertheless, when two different sources of HT (TA; 1.5 g/100 g DM or chestnut; 2 g/100 g DM; both contained 1.43 g/100 g HT in dietary DM) and a subunit of the HT (GA; 1.5 g/100 g DM) were added to a forage-based diet, the subunit of HT (in the form of GA) decreased both CH_4_ yield and the proportion of gross energy intake emitted as CH_4_ (by 9% compared with the control; [8]). Additionally, there were no negative effects on nutrient digestibility, including that of CP with GA addition, but it decreased urea and uric acid in urinary N compared with the control [8]. These results suggest that GA may be toxic to some microbes [58], thereby reducing CH_4_ production and improving N utilization of ruminants. However, it is not known whether GA or its metabolites have negative effects on animal performance.

A meta-analysis from 15 in vivo experiments showed that a linear decrease in CH_4_ production expressed relative to DMI or digestible OM intake with increasing tannin concentration [11]. The study reported a decrease of 0.011 L CH_4_/100 g DMI or 0.012 L CH_4_/ 100 g digestible OM for each g/ 100 g of tannin in the diet (r^2^ = 0.47 or 0.29, respectively). However, some of the CH_4_ decrease was due to the concomitant decline in digestibility of OM, especially fibre. A reduction in the intake of digestible OM would negatively affect animal performance. Another major limitation with the use of tannins to mitigate CH_4_ production is that at low concentrations (<2.0 g/100 g DM), typical of many forages and feed supplements, CH_4_ responses are highly variable. This effect is partly due to the binding ability of tannin to dietary nutrients. At low concentrations of tannins, there are insufficient free tannins to directly inhibit methanogens because other dietary components, such as fibre and protein, are easily bound to the free tannins. Therefore, tannins present in low concentrations in forages or extracts used as dietary supplements may not produce a consistent reduction in CH_4_ production.

In the same meta-analysis [11], an increase in DMI with increasing tannin concentration was reported; however, other studies as reviewed by Waghorn [28] reported the opposite trend. The relationship between tannin concentration and DMI is confounded by the digestibility of the forage, and numerous factors that affect intake. The relationship may also differ depending on the type of ruminant species. For instance, goats are commonly fed tropical pastures with high tannin concentration and they have been shown to more easily adapt to tannin-containing feeds than sheep and cattle. Salivary proteins (proline or histatin) bind to tannins, thereby causing astringency, but such interactions can also act as a defensive mechanism against the potential negative effects of tannin consumption. The higher production of tannin-binding salivary proteins in goats makes them less susceptible to the negative effects of tannins relative to sheep and cattle [90]. In a study by Liu et al. [83], goats were fed *Lespedeza cuneate* with quebracho extract at a CT level of 7.5 to 9 g/100 g of the diet without any negative effect on performance, although nutrient digestibility decreased and CH_4_ production also decreased by 54% to 58% relative to an alfalfa based diet.

The small number of in vivo studies that have been conducted using HT suggests a more consistent reduction of CH_4_ production from ruminants compared with CT (Table 4). However, the optimum level of HT or its subunit, GA, in decreasing CH_4_ production from ruminants is not known. It is possible that gradual adaptation of sheep and cattle to HT may allow them to consume diets containing >2.0 g HT/ 100 g DM with no negative effects on animal performance while decreasing their environmental impact.

## 5. Conclusions

In conclusion, there is sufficient information to indicate the potential of using terrestrial plant tannins to mitigate enteric CH_4_ emissions from forage-fed ruminants. When examined overall, there is indication that higher MW tannins, CT, lack consistent effects on enteric CH_4_ reduction, and some of the mitigation effect may be due to a decrease in DMI or diet digestibility. However, several recent studies have suggested that lower MW tannins, HT, may reduce enteric CH_4_ emission without negative effects on digestibility, with effects attributed to the GA subunit or its metabolites. There is a need to understand the effect of low MW and GA-containing tannins and their metabolites on methanogens. Research on the effects of tannin for CH_4_ mitigation is at an early stage and warrants further investigation. The use of tannin-containing diets to reduce CH_4_ emissions is of great interest for grazing ruminants and developing countries where limited mitigation options are available. The optimum concentration and sources of tannin for decreasing CH_4_ production without adverse effect on animal performance needs further study.

## Figures and Tables

**Table 1:** Summary of the concentration and main type of tannins in some temperate and tropical forages.

Source	Forages	Tannin ^1^	
		(g/100 g DM)	Type
	**Legumes (temperate)**		
Terrill et al. [32], Jackson et al. [33]	Birdsfoot trefoil (*Lotus corniculatus*)	0.7 to 4.0	CT
Terrill et al. [32]	Crownvetch (*Coronilla varia*)	1.6	CT
Terrill et al. [32], Schreurs et al. [34]	Greater birdsfoot trefoil (*Lotus pedunculatus*)	6.1 to 9.9	CT
Berard et al. [35]	Purple prairie clover (*Dalea purpurea*)	3.8 to 9.3	CT
Jackson et al. [33], Berard et al. [35]	Red clover (*Trifolium pratense*)	0.04 to 1.53	CT
Berard et al. [35], McMahon et al. [36]	Sainfoin (*Onobrychis viciifolia*)	1.6 to 9.4	CT
Terrill et al. [32]	Serradella (*Ornithopus sativus)*	0.4	CT
Terrill et al. [32], Jackson et al. [33], Waghorn et al. [37]	Sulla (*Hedysarum coronarium*)	3.3 to 6.8	CT
Schreurs et al. [34], Berard et al. [35]	White clover (*Trifolium repens*)	0.1 to 1.2	CT
	**Legumes (tropical)**		
Jackson et al. [33], Hove et al. [38]	Calliandra (*Calliandra calothyrsus*)	11.6 to 19.6	CT
Priolo et al. [39], Silanikove et al. [40]	Carob tree (*Ceratonia silique*)	3.0 to 17.0	CT
Jackson et al. [33], Barahona et al. [41]	Desmodium (*Desmodium ovalifolium*)	9.4 to 23.8	CT
Jackson et al. [33], Hove et al. [38]	Leucaena (*Leucaena leucocephala*)	5.4 to 13.4	CT
Smith et al. [42], Norton [43]	White ball acacia (*Acacia angustissima*)	0.7 to 17.4	CT
	**Trees (Tropical)**		
Gemeda and Hassen [44]	African milkbush (*Euphorbia tirucalli*)	7.6	HT
Gemeda and Hassen [44]	African sumac (*Rhus lancea*)	13.9	HT
Tahrouch et al. [45]	Argan tree (*Argania spinose*)	14.0	CT
Gemeda and Hassen [44]	Northern red oak (*Quercus rubica*	8.8	HT
Gemeda and Hassen [44]	Sacred fig (*Ficus religiosa*)	9.3	HT

^1^ Tannin concentration in the plant or tannin extract concentration in the substrate; DM = dry matter; CT = condensed tannin; HT = hydrolysable tannin.

**Table 2:** Summary of potential nutritional and toxicity effects of tannins in ruminants.

Source	Tannin			Effect
	Plant/Extract	Type ^1^	g/100 g DM ^2^	
				**Beneficial**
Min et al. [48]	Chestnut and mimosa extracts	HT and CT, respectively	0.0 to 1.5	Decreased the number of days that heifers experienced bloat by 81% and 77%, respectively, compared with control (no tannin diet). Increased average daily gain of heifers by 20% and 6%, respectively, compared with the control animals.
Martínez-Ortíz-de-Montellano et al. [49]	Tzalam (*Lysiloma latisiliquum*)	CT	5.5	Worm fecal egg count decreased by 33% for lambs fed *L. latisiliquum* compared with those fed control diet (no tannin) after day 36 of dosing lambs with *Haemonchus contortus.*
Aboagye et al. [7]	Chestnut and quebracho extracts	HT and CT, respectively	0.0 to 1.5	Rumen ammonia N decreased by 44% for beef cattle fed tannin supplements compared with the control (no tannin diet)
Aboagye et al. [8]	Tannic acid, chestnut and gallic acid	HT sources and HT subunit, respectively	0.0 to 2.0	Tannic acid and chestnut increased the proportion of N excreted in feces and decreased the proportion in urine in growing beef cattle compared with control animals (43.9% vs. 37.8% and 56.1% vs. 62.2%; respectively).
Woodward et al. [50]	Birdsfoot trefoil (*Lotus corniculatus*)	CT	2.6	*Lotus corniculatus* increased milk production by 33% in dairy cattle compared with those fed ryegrass (no tannin). Methane production per unit of DMI also decreased by 17% for dairy cattle fed *L. corniculatus* relative those fed ryegrass.
				**Negative**
Dschaak et al. [51]	Quebracho extracts	CT	0.0 or 3.0	Supplementing tannin decreased DMI by 6% in dairy cows fed either a high forage or low forage diet.
Henke et al. [52]	Quebracho extracts	CT	0.0, 1.5, or 3.0	There was no negative effect with tannin added at 1.5 g/100 g DM, but at 3.0 g/100 g DM, tannin decreased nutrient digestibilities with greater effect on crude protein digestibility; and so, milk yield, milk fat and protein contents decreased for dairy cows.
Garg et al. [53]	Oak (*Quercus incana*)	HT and CT mixture	9.8 and 0.6, respectively	Cattle fed *Q. incana* had anorexia, severe constipation and brisket edema with 70% mortality.
Robins and Broker [54]	Mulga (*Acacia aneura*) or oaten hay chaff	CT	7.5 and 0.03 respectively	In sheep fed a *A. aneura* diet, DMI and body weight were reduced with tissue fragility at discrete areas of the abomasum compared with sheep fed the oaten hay chaff.

^1^ CT = condensed tannin; HT = hydrolysable tannin. ^2^ Tannin concentration in the plant or tannin extract concentration in the substrate.

**Table 3:** Summary of tannin effects on in vitro fermentation, degradability, microbes and enteric methane emission in ruminants.

Source	Animal (Rumen Fluid)	Forage Substrate and Level ^1^	Tannin				Effects ^5^				
			Plant/Extract	Type ^2^	g/100 g ^3^	Molecular weight ^4^	VFA	NH_3_	CH_4_ Yield ^6^	Degradability	Microbes
Jayanegara et al. [10]	Cattle	Hay:concentrate (0.38 g; 70:30).	Extracts of chestnut, sumach, mimosa, quebracho	HT, HT, CT, CT, respectively	0.0 to 1.0 mg/mL	NR but HT < CT	↓ except sumach	NR	↓ (4.3 % for HT and 2.5% for CT).	↓ OM	↓ methanogens (only for the 1 mg/mL).
Nauman et al. [15] Nauman et al. [71]	Cattle	Same plants (0.2 g; 100%)	*Leucaena retusa*, *Desmanthus illinoensis*, *Neptunia lutea, Acacia angustissima*, *Lespedeza stuevei,* and *Desmodium paniculatum*	CT	3.3, 8.2, 8.3, 8.7, 11.7 and 12.5, respectively	1745 Da, 1369 Da, 3025 Da, 1241 Da, 1473 Da and 2065 Da, respectively	↓ except for *L. retusa*	NR	↓ (70% relative to *Arachis glabrata*, 0.6% CT) except for *L. retusa*	NR	NR
Gemeda and Hassen [44]	Sheep	Same plants (0.4 g ± PEG; 100%).	*Melia azedarach*, *Peltrophorum africanum*, *Rhus lancea*	Mixture of HT and CT	HT = 0.68 to 13.9; CT = 0.65 to 6.0	NR	↓	↓	↓ (59%)	↓ OM	NR
Hassanatand Benchaar [72]	Cattle	Forage:concentrate (65:35; 0.2 g)	Acacia, quebracho; and chestnut, valonea extracts	CT; and HT, respectively	0.0 to 20.0	NR	↓ except for valonea at 50 g/kg DM	↓	↓ (40 relative to control	NR	NR
Mengistu et al. [73]	Goat	Same plants (0.5 g ± PEG; 100%).	*Euclea racemose, Rhus natalensis, Maytenus senegalensis*	CT	> 20.0	NR	↓	↓	↓ (42%)	↑ OM	NR
Pellikaan et al. [74]	Cattle	Alfalfa (0.4 g ± PEG; 100%)	Green tea, quebracho, grape seed; and chestnut, valonea myrabolan, tara extracts	CT; and HT, respectively	CT = 6.6 to 17.0; HT = 2.9 to 19.0	CT = 481.8 to 2237.4 Da; HT = 655.5 to 2191.0 Da	↓	↓	↓ (21%)	NR	NR
Rira et al. [75]	Sheep	*Dichanthium spp* (0.4 g; 100%)	*Acacia nilotica (leaves or pods)*	HT	17.8 to 35.0	NR	↓	NR	↓ (55 to 64%)	↑ DM for leaves and pods	NR
Saminathanet al. [76], Saminathanet al. [77]	Cattle	Guinea grass (0.5 g; 100%)	*Leucaena leucocephala* extract	Unfraction-ated CT (F0) Fractionat-ed CT (F1 to F5)	0.0 and 3.0	F0 = 1293.0 Da; F1 = 1265.8 Da; F2 = 1028.6 Da; F3 = 652.2 Da; F4 = 562.2 Da; F5 = 469.6 Da.	↓ For F0 and F1	NR	↓ for all MW of CT (i.e., F0 to F5; average = 28%).	− DM; ↓N for only F1.	↓ total methanogens with increasing MW but in relative abundance, the rumen cluster C was the most abundant archaeal community and it ↑ with increasing MW of CT.
Soltan et al. [78]	Sheep	Same plants (0.5 g; 100%).	*Acacia saligna,* and *Leucaena leucocephala*	CT	6.3 and 4.6, respectively	NR	−	−	↓ (37% relative to Tifton hay, 0% tannin)	↑ undigested ruminal protein compared with Tifton hay	↓ protozoa relative to Tifton hay
Tan et al. [79]	Cattle	Guinea grass (0.5 g; 100%)	*Leucaena leucocephala* extract	CT	0.0 to 6.0	NR	↓ with increasing CT dosage	NR	↓ with increasing CT dosage (average = 52%).	↓DM and N with increasing CT dosage.	↓methanogens and protozoa with increasing dose of CT.
Tavendale et al. [80]	Sheep	Same plant (0.5 g ± PEG; 100%).	*Lotus pedunculatus*	CT	10.0	NR	−	↓	↓ (20%)	NR	Oligomeric fractions were inactive against *Methanobrevibacter ruminantium* relative to polymeric fraction in broth culture.

^1^ Same plant is where a tannin-containing forage was used as the forage substrate; dietary level is on dry matter (DM) basis; PEG = polyethylene glycol (binds to tannin and acts as a control) ^2^ CT = condensed tannin; HT = hydrolysable tannin. ^3^ Tannin concentration in the plant or tannin extract concentration in the substrate; unit is the same unless otherwise specified. ^4^ NR = not reported; F0 to F5 = fractions of molecular weight from highest to lowest. ^5^ ↑ = increase; ↓ = decrease; ─ = no statistically significant effect; NR = not reported; MW = molecular weight; OM = organic matter. ^6^ CH_4_ yield = g CH_4_/ g DM degraded, g CH_4_/ g OM degraded, or g CH_4_/ g DM incubated. ↓ ↓.

**Table 4:** Summary of tannin effects on in vivo fermentation, digestibility, microbes and enteric methane production in ruminants.

Source	Animal (rumen fluid)	Forage substrate and level ^1^	Tannin			Effects ^4^				
			Plant/Extract	Type ^2^	g/100 g DM ^3^	VFA	NH_3_	CH_4_ yield ^5^	Digestibility	Microbes
Aboagye al. [7]	Cattle	Alfalfa silage:barley silage (50:50; 95%)	Chestnut and Quebracho extracts	HT and CT, respectively	0.0 to 1.5	−	↓	↓ (6% for 1.5% HT and CT combination).	NR	− protozoa
Aboagye al. [8]	Cattle	Alfalfa silage:barley silage (79:21; 95%)	Tannic acid chestnut and gallic acid	HT sources and HT subunit, respectively	0.0 to 2.0	↑for only gallic acid	↓ for only tannic acid	↓ (9 % for gallic acid).	− nutrients, except ↓crude protein for HT sources	− protozoa
Ebert et al. [81]	Cattle	Sorghum stalk: concentrate (8.5:91.5)	Quebracho extract	CT	0.0, 0.5, or 1.0	NR	NR	−	− DM and OM	NR
Lima et al. [82]	Sheep	Elephant grass: concentrate (60:40).	*Mimosa tenuiflora* extract	CT	0.0 and 3.0	NR	NR	−	− nutrients	↓protozoa
Liu et al. [83]	Goat	Forage: concentrate (75:25).	*Lespedeza cuneate* with quebracho extract	CT	7.5 to 9.0	↓	−	↓ (54 to 58% relative to a control, i.e., an alfalfa based diet).	↓ for all nutrients	− bacteria but ↓protozoa
Malik et al. [84]	Sheep	Forage: concentrate (60:40).	*Artocarpus heterophyllus, Azadirachta indica and Ficus benghalensis*	CT	7.2 to 10.9	↓	−	↓ (24% relative to wheat bran control diet).	↓ DM for *Azadirachta indica* relative to the other tannin-containing and control (no tannin) diets.	− bacteria but ↓protozoa
Stewart et al. [85]	Cattle	Same plants (100%)	Birdsfoot trefoil, sainfoin and small burnet	CT, CT and HT respectively	0.6, 2.5 and 4.5, respectively	NR	NR	↓ (39% for HT relative to CT).	Sainfoin and small burnet ↓nutrients and crude protein relative to birdsfoot trefoil.	NR
Supapong et al. [86]	Cattle	Rice straw: concentrate (80:20)	*Delonix regia* seed meal	CT	0.0, 9.0, 18.0 or 27.0	−	↑ with increased CT	↓ (16% relative to no tannin).	↓DM and OM with increasing CT concentration.	↓protozoa
Yang et al. [87]	Cattle	Corn silage: concentrate (50:50)	Tannic acid	HT	0, 0.65, 1.3 or 2.6	↓	↓	↓ (11, 15, and 34%, respectively relative to no tannin)	↓DM, OM and protein.	↓protozoa and methanogens (only for the 2.6% DM).

^1^ Same plant is where a tannin-containing forage was used as the forage substrate; dietary level on a dry matter (DM) basis. ^2^ CT = condensed tannin; HT = hydrolysable tannin; Molecular weight was not measured in any of these studies but HT < CT and gallic acid < tannic acid. ^3^ Tannin concentration in the plant or tannin extract concentration in the diet. ^4^ ↑ = increase; ↓ = decrease; ─ = no statistically significant effect; NR = not reported; OM = organic matter. ^5^ CH_4_ yield = g CH_4_/ g DM degraded or g CH_4_/ g OM degraded.
